# Enzymatic properties of CARF-domain proteins in *Synechocystis* sp. PCC 6803

**DOI:** 10.3389/fmicb.2022.1046388

**Published:** 2022-11-07

**Authors:** Jin Ding, Nils Schuergers, Heike Baehre, Annegret Wilde

**Affiliations:** ^1^Molecular Genetics of Prokaryotes, Institute of Biology III, University of Freiburg, Freiburg, Germany; ^2^Research Core Unit Metabolomics, Hannover Medical School, Hannover, Germany

**Keywords:** CRISPR, cyclic oligoadenylate signaling, CARF, HEPN, cyanobacteria

## Abstract

Prokaryotic CRISPR-Cas (clustered regularly interspaced short palindromic repeats and CRISPR-associated genes) systems provide immunity against invading genetic elements such as bacteriophages and plasmids. In type III CRISPR systems, the recognition of target RNA leads to the synthesis of cyclic oligoadenylate (cOA) second messengers that activate ancillary effector proteins *via* their CRISPR-associated Rossmann fold (CARF) domains. Commonly, these are ribonucleases (RNases) that unspecifically degrade both invader and host RNA. To mitigate adverse effects on cell growth, ring nucleases can degrade extant cOAs to switch off ancillary nucleases. Here we show that the model organism *Synechocystis* sp. PCC 6803 harbors functional CARF-domain effector and ring nuclease proteins. We purified and characterized the two ancillary CARF-domain proteins from the III-D type CRISPR system of this cyanobacterium. The Csx1 homolog, SyCsx1, is a cyclic tetraadenylate(cA4)-dependent RNase with a strict specificity for cytosine nucleotides. The second CARF-domain protein with similarity to Csm6 effectors, SyCsm6, did not show RNase activity *in vitro* but was able to break down cOAs and attenuate SyCsx1 RNase activity. Our data suggest that the CRISPR systems in *Synechocystis* confer a multilayered cA4-mediated defense mechanism.

## Introduction

The majority of archaeal and almost half of all bacterial genomes encode CRISPR-Cas (clustered regularly interspaced short palindromic repeats and CRISPR-associated genes) systems which provide immunity against invading genetic elements such as bacteriophages or plasmids (Makarova et al., 2020a). These systems acquire short sequences of foreign DNA that are integrated as new spacers between repeat sequences of CRISPR loci. Once transcribed and matured, CRISPR RNAs (crRNA) serve as guides for effector complexes composed of a single or multiple Cas protein(s) that can recognize and degrade foreign nucleic acids (Sorek et al., 2013; Marraffini, 2015; Mohanraju et al., 2016). CRISPR-Cas systems, which show an exceptional diversity in gene composition and modular organization, can be classified into two classes with six distinct types (Types I–VI) and over 30 subtypes (Makarova et al., 2015, 2020a). Intriguingly, a single organism can contain multiple and diverse CRISPR-Cas systems.

The hallmark of the widespread Type III CRISPR-Cas systems is the presence of the large subunit Cas10 in the effector complex that targets both invader RNA transcripts and invader DNA. Most Cas10 proteins have two enzymatic activities related to the defense function of CRISPR systems. A histidine-aspartate (HD) nuclease domain was shown to provide immunity by degrading single-stranded target DNA in a transcription-dependent manner (Samai et al., 2015; Elmore et al., 2016; Kazlauskiene et al., 2016). Moreover, using ATP as substrate, a pair of composite Palm domains in Cas10 generates cyclic oligoadenylates (cOAs) with various ring sizes, containing between three and six 3′–5′ linked AMP units (cA3-cA6). This polymerase/cyclase activity depends on an intact GGDD motif in the Palm domain and is inhibited as soon as the target RNA is degraded (Kazlauskiene et al., 2017; Niewoehner et al., 2017; Rouillon et al., 2018).

The cOA molecules constitute key second messengers which activate Type III ancillary proteins, typically Csm6 or related Csx1 family RNases. These RNases harbor a CRISPR-associated Rossmann fold (CARF) nucleotide-binding domain, which upon binding of the cOA ligand, allosterically activates the promiscuous RNase activity of a nucleotide-binding (HEPN) effector domain (Kazlauskiene et al., 2017; Niewoehner et al., 2017). Indiscriminate RNA degradation of host and invader transcripts constitutes an additional interference mechanism that boosts immunity and invader DNA clearance by preventing invader replication, arresting cell growth, and inducing cell dormancy or cell death (Jiang et al., 2016; Foster et al., 2019; Rostøl and Marraffini, 2019). To switch off these RNases and limit self-toxicity, cOA must be removed from the cell once the invading genetic elements have been cleared. Several organisms evolved ring nucleases which are CARF domain containing enzymes that cleave the cOA messenger in a metal-independent mechanism and convert it into two molecules of di-adenylate containing a 2′,3′-cyclic phosphate (A2 > P; Athukoralage et al., 2018; Molina et al., 2019). However, not all prokaryotes that harbor Type III CRISPR-Cas systems encode a dedicated CARF domain ring-nuclease. Several organisms employ dual-function RNases with a CARF domain that acts as a cOA sensor and has ring-nuclease activity (Athukoralage et al., 2019; Jia et al., 2019b; Garcia-Doval et al., 2020; Smalakyte et al., 2020). Others encode homologs of Csx3 in their CRISPR III ancillary modules. This Mn-dependent exoribonuclease has a structure which is distinct from the Rossmann fold and has been characterized as a ring nuclease that degrades cA4 (Athukoralage et al., 2020a).

**Figure 1:**
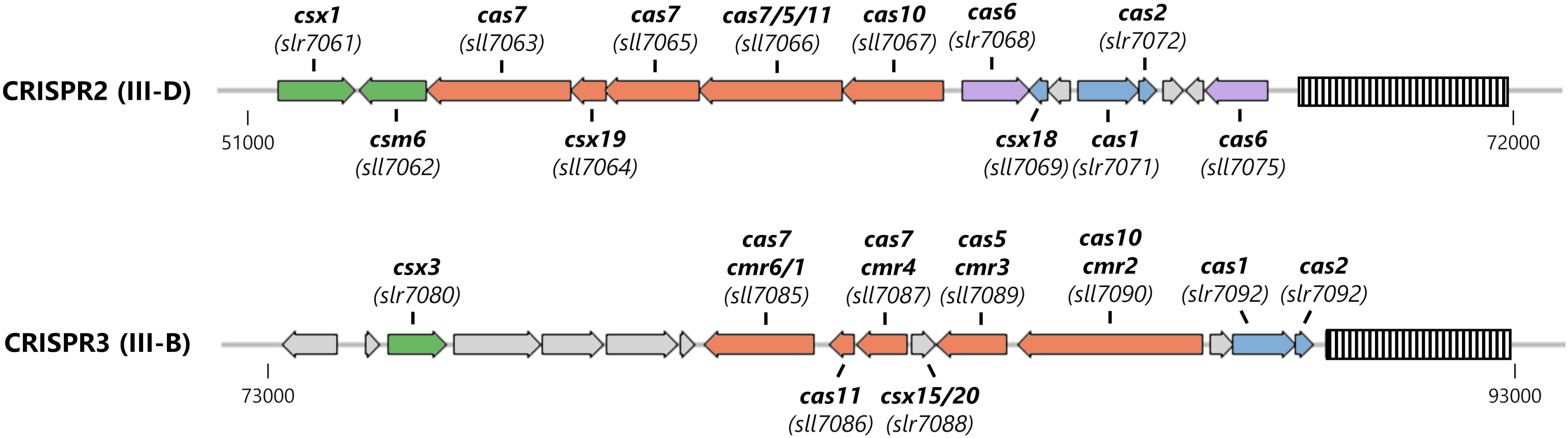
Organization of the Type III CRISPR-Cas systems encoded on the large plasmid pSYSA in *Synechocystis*. The CRISPR arrays are shown as striped boxes. Genes are color-coded: adaptation-associated genes (blue), effector complex components (salmon), pre-crRNA processing (purple), and ancillary genes putatively involved in cOA-signaling (green).

About two thirds of all sequenced cyanobacterial genomes encode a CRISPR-Cas system (Makarova et al., 2020a). The model organism *Synechocystis* sp. PCC 6803 (from here on *Synechocystis*) contains three CRISPR-Cas systems (named CRISPR1-3), which are encoded on the stably transmitted plasmid pSYSA (Figure 1). With the exception of CRISPR2 which has not been characterized in detail, these systems were shown to efficiently mediate immunity against invading plasmids (Behler et al., 2018; Shah et al., 2019). According to their gene composition, CRISPR1 can be classified as a I-D system, whereas CRISPR2 and CRISPR3 are type III-D and type III-B systems, respectively harboring two Palm domains in the large effector complex subunits (Hein et al., 2013; Scholz et al., 2013; Reimann et al., 2017). Adjacent to the Type III-D interference complex, two accessory genes encoding CARF domain proteins have been identified (Shah et al., 2019). The last ORF (*sll7062*) in the CRISPR2 effector complex operon [transcriptional unit TU7058 (Kopf et al., 2014)] encodes a Csm6 family protein. This Csm6 homolog (hereafter SyCsm6) has an N-terminal CARF domain of the CARF7 family fused to a RelE domain, a nonspecific RNase found in numerous toxin-antitoxin systems (Griffin et al., 2013; Makarova et al., 2020b). This domain architecture is strongly linked to Type III CRISPR-Cas systems (Shah et al., 2019). Next to *sll7062* on the opposite strand, *slr7061* encodes a Csx1 family protein (SyCsx1) with a CARF-HEPN domain structure, the most abundant ancillary protein architecture associated with Type III (A, B, or D) CRISPR–Cas systems (Shah et al., 2019; Makarova et al., 2020b). Furthermore, a *csx3* homolog (*slr7080*) is encoded upstream of the CRISPR3 module. Deletion of the putative *csx3* gene did not affect the ability of the system to defend against an invader plasmid (Shah et al., 2019).

Considering the presence of these genes, we hypothesize that *Synechocystis* harbors a functional cOA-signaling pathway. Therefore, we heterologously expressed and purified the CRISPR-associated CARF-domain proteins from *Synechocystis* to characterize their enzymatic properties. We show that SyCsx1, but not SyCsm6, has a cA4-dependent RNase activity *in vitro*. However, SyCsm6 was able to degrade cOA and abolished the RNase activity of SyCsx1. Our results suggested that both CARF-domain proteins orchestrate cOA-dependent ribonucleolytic activity in tandem.

## Results

### SyCsx1 is a cA4-activated RNase with a preference for cytosines

**Figure 2:**
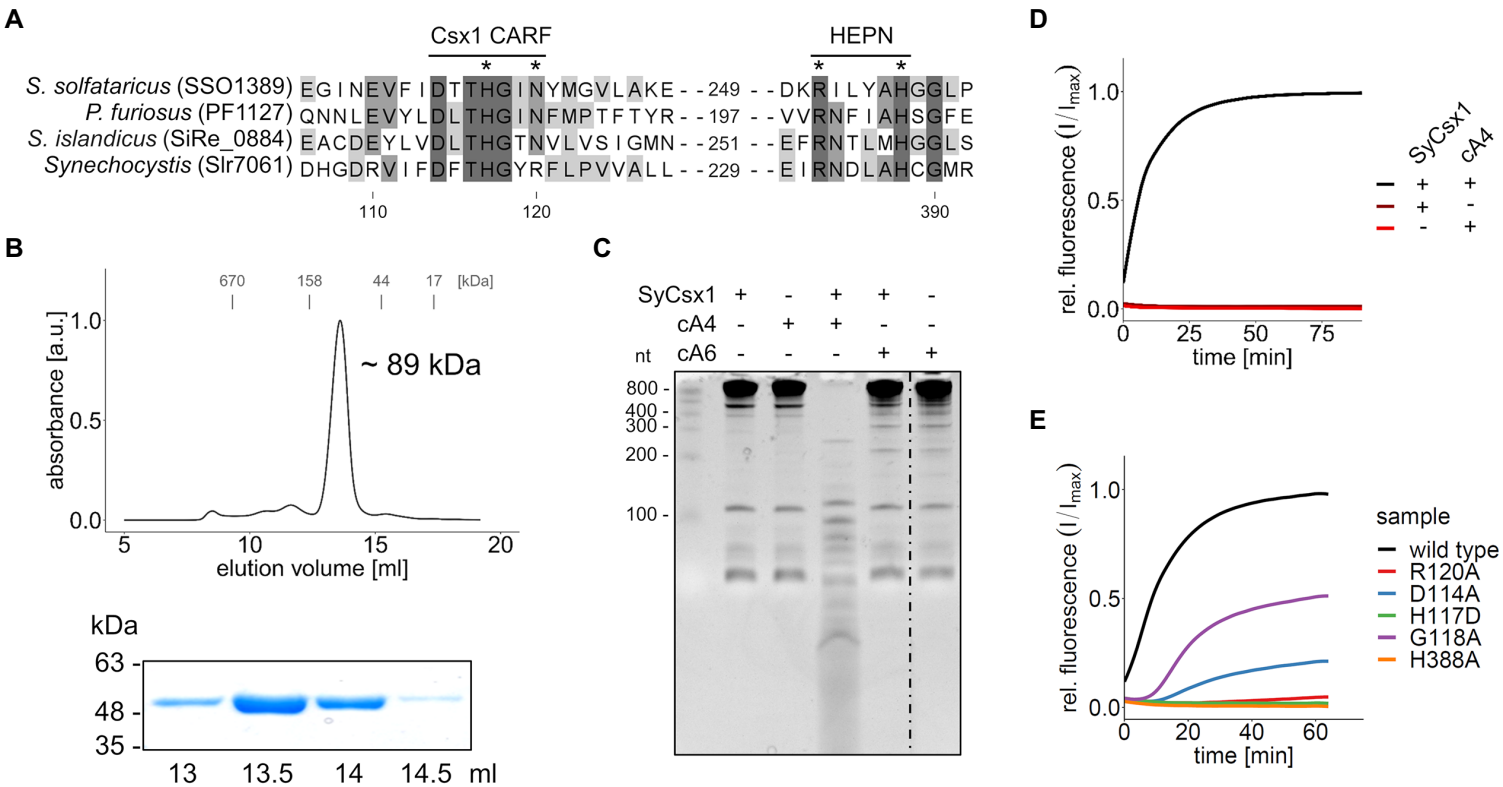
SyCsx1 shows cA4-dependent ribonuclease activity. **(A)** Multiple sequence alignment of SyCsx1 and homologous Csx1 proteins showing the conserved DxTHG motif from the CARF domain and the RxxxxH active site of the HEPN domain. An asterisk marks residues that were shown to impact protein function. **(B)** His-tagged SyCsx1 (48.7 kDa) was isolated from *E. coli* by affinity chromatography and further purified by size-exclusion chromatography on a Superdex 200 10/300 column (upper panel). Elution fractions were analyzed by SDS-PAGE (lower panel, see Supplementary Figure S1). **(C)** 400 ng of *Synechocystis* total RNA were incubated in the presence or absence of SyCsx1 and 300 nM cOAs for 1 h and subsequently separated on a 12% denaturing PAA gel. The dotted line indicates a non-contiguous sample that was omitted from the gel. **(D)** A fluorogenic ribonuclease activity assay measuring the cleavage of 40 nM RNaseAlert substrate by SyCsx1 depending on the addition of 300 nM cA4 (excitation 490 nm; emission 520 nm). The cleavage of the RNaseAlert substrate separates a fluorophore/quencher pair, generating a fluorescent signal. **(E)** Fluorogenic ribonuclease activity assay of SyCsx1 variants with point mutations in the ligand-binding site of the CARF domain or the active site of the HEPN domain. All fluorogenic assays were performed in triplicates and mean values of relative fluorescence normalized to the maximum substrate turnover are shown.

The gene *csx1* from the *Synechocystis* CRISPR2 locus (Figure 1) encodes a CARF-domain protein whose function has not yet been elucidated. Sequence comparison to biochemically characterized Csx1 homologs showed that the distinct DxTHG motive, which is part of the ligand-binding surface of the CARF domain (Makarova et al., 2020b) and the HEPN active site residues (RXXXXH; R-X4-6-H) (Anantharaman et al., 2013) are conserved in SyCsx1 (Figure 2A). To confirm that SyCsx1 functions as a cOA-dependent RNase, we expressed and purified recombinant His-tagged SyCsx1 (49.3 kDa). Size exclusion chromatography revealed a monodisperse peak at around ~89 kDa (Figure 2B), suggesting that SyCsx1 forms a dimer in solution. Dimerization is typically observed in solved CARF-domain protein structures and is important for cOA binding and RNA decay (Kim et al., 2013; Molina et al., 2019). We tested RNase activity of SyCsx1 on *Synechocystis* total RNA in the presence or absence of cA4 and cA6, which are the most common CARF-domain ligands that activate effector domains (Athukoralage and White, 2021). Analysis of RNA cleavage by denaturing polyacrylamide (PAA) gel electrophoresis (Figure 2C) demonstrated that SyCsx1 without a specific activator does not degrade total RNA. Only if it is stimulated by the second messenger cA4, but not by cA6, it shows RNase activity that leads to a substantial degradation of total RNA. Additionally, we assayed the cOA-dependent cleavage of a fluorogenic RNA substrate (Figure 2D). SyCsx1 is clearly inactive in the absence of cOAs, while the addition of cA4 leads to a significant increase of the fluorescence signal and presumably the complete turnover of the substrate.

To confirm the role of the ligand-binding CARF and the HEPN effector domain for SyCsx1 function, we performed RNase activity assays with recombinant protein variants in which we exchanged conserved residues in the CARF and HEPN domains (Figure 2E). In the presence of cA4, mutations in the conserved DxTHG motif (D114A, G118A) attenuated RNase activity and replacement of residues (H117D, R120A) that are known to participate in cOAs binding in other CARF domain proteins (Molina et al., 2019), reduced RNA cleavage to barely measurable levels. Furthermore, mutating an active site residue of the conserved HEPN-associated RxxxxH motif (H388A) completely abolished RNase activity of SyCsx1. Taken together, these results confirm that cA4-binding by the CARF domain allosterically regulates the nuclease activity of the C-terminal HEPN domain in SyCsx1.

**Figure 3:**
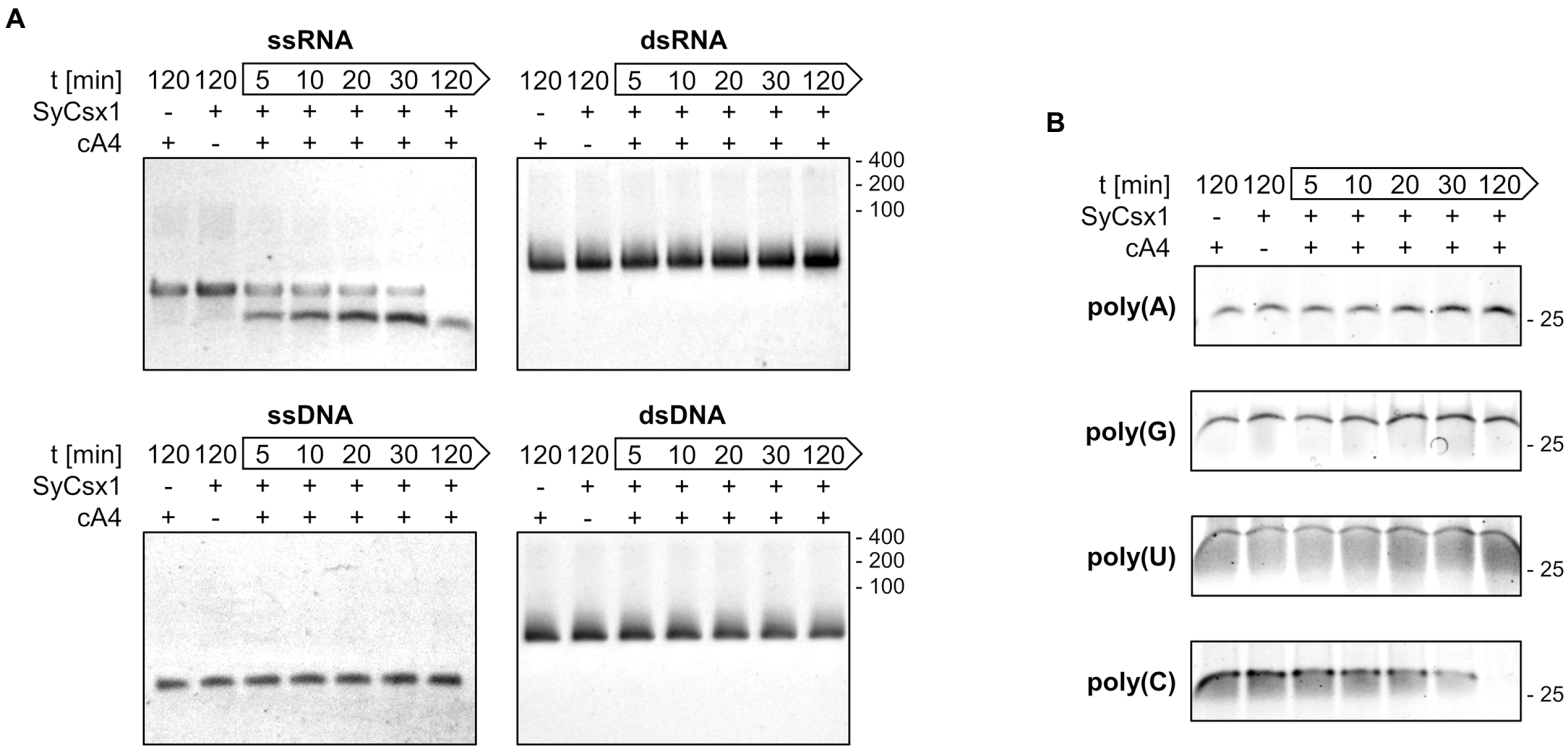
SyCsx1 is a single-strand-specific ribonuclease with a strict preference for cytosine bases. **(A)** Cleavage of 5 μM single- and double-stranded RNA or DNA oligonucleotides (40 nt) by SyCsx1 at 30°C after the addition of 300 nM cA4. 10 μl aliquots of were sampled at indicated time points, separated on 12% denaturing PAA gels, and stained with ethidium bromide. **(B)** 25 nt RNA-homopolymers were incubated with SyCsx1 and cA4 at 30°C and analyzed as before, except that SybrGold was used for staining.

To narrow down the substrate specificity of SyCsx1, we analyzed the cleavage of single (ss) and double-stranded (ds) RNA and DNA substrates (Figure 3A). Denaturing PAA gel electrophoresis revealed that in the presence of cA4 only the ssRNA substrate but neither dsRNA nor the DNA substrates were degraded. The accumulation of a distinct ssRNA cleavage product hints at a sequence-specific cleavage mechanism. To analyze the base specificity of SyCsx1, we performed cleavage assays with the 25mer homooligonucleotides poly(A/U/C/G). SyCsx1 degraded poly(C) completely within 120 min, while the other three homopolymers were stable in the presence of the protein (Figure 3B). These results demonstrate that Csx1 is a cA4-dependent ssRNA RNase with strict specificity for cytosine nucleotides.

### SyCsm6 does not show RNase activity *in vitro*

**Figure 4:**
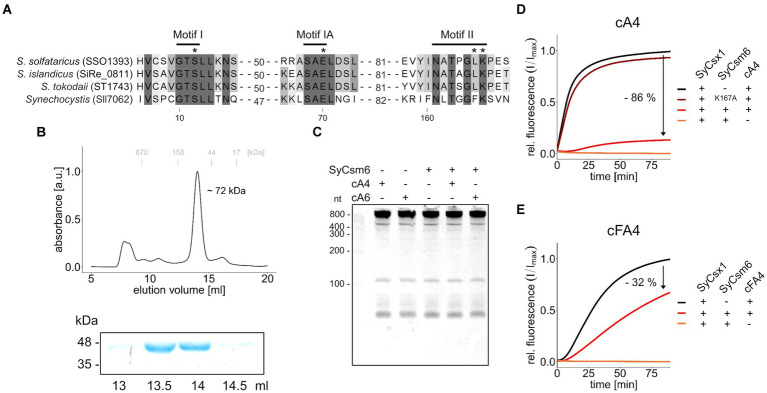
SyCsm6 has ring-nuclease activity but does not cleave RNA *in vitro***. (A)** Multiple sequence alignment of SyCsm6 (Sll7062) and homologous Csm6/Crn1 proteins showing conserved CARF domain motives implicated in cOA-binding and cleavage. An asterisk marks residues that were shown or predicted to impact ring nuclease activity. **(B)** His-tagged SyCsm6 (43.5 kDa) was isolated from *E. coli* by affinity chromatography and further purified by size-exclusion chromatography on a Superdex 200 10/300 column (upper panel). Elution fractions were analyzed by SDS-PAGE (lower panel, see Supplementary Figure S1). **(C)** 400 ng of *Synechocystis* total RNA were incubated in the presence or absence of SyCsm6 and 300 nM cOAs for 1 h and subsequently separated on a 12% denaturing PAA gel. **(D)** A fluorogenic ribonuclease activity assay measuring RNA cleavage by SyCsx1 after pre-treatment of 30 pmol cA4 with either wild-type SyCsm6, the putatively inactive K167A variant, or a control without protein. After the pre-treatment for 60 min at 30°C and inactivation of SyCsm6 by heating to 95°C for 5 min, the reaction was started by adding SyCsx1 and 20 nm RNaseAlert substrate (excitation 490 nm; emission 520 nm). **(E)** The same assay as before using tetrafluoro-c-tetraAMP (cFA4) as the substrate. All fluorogenic assays were performed in triplicates and mean values of relative fluorescence normalized to the maximum substrate turnover are shown.

The other CARF domain protein found in *Synechocystis* is SyCsm6. The respective ORF *sll7062* is transcribed as part of the CRISPR2 effector complex operon (Kopf et al., 2014). To determine enzymatic activity, recombinant His-tagged SyCsm6 was expressed and purified from *Escherichia coli*. In size exclusion chromatography, SyCsm6 (43.5 kDa) eluted at a volume corresponding to around ~72 kDa (Figure 4B), suggestive of dimer formation similar to SyCsx1 and other CARF domain proteins. First, we performed cleavage assays of purified SyCsm6 with *Synechocystis* total RNA in the presence or absence of cA4 and cA6. Separation of the reaction products on denaturing PAA gels did not reveal any degradation of total RNA (Figure 4C), implying that SyCsm6 does not possess RNase activity under the tested conditions. However, SyCsm6 does not encompass a HEPN domain at its C-terminus but a putative RelE-like effector domain. The *E. coli* toxin RelE and homologous proteins are ribosome-dependent endoribonucleases that inhibit translation, leading to growth arrest (Pedersen et al., 2003). Therefore, it is conceivable that SyCsm6 can degrade actively translated mRNA in a cOA-dependent manner *in vivo*.

### SyCsm6 is a ring nuclease degrading cOA

Sequence- and structure-based classification revealed that the CARF domain of SyCsm6 is similar to the corresponding domains of CRISPR ring nucleases 1 (Crn1) from crenarchaea of the genus *Sulfolobus* (Makarova et al., 2020b). A direct sequence comparison (Figure 4A) shows that critical residues in two motives implicated in ligand-binding (K167) and ring-nuclease activity (E70, S12) of cOA-degrading CARF-domain proteins are present in *Synechocystis* SyCsm6 (Athukoralage et al., 2018, 2019; Garcia-Doval et al., 2020; Makarova et al., 2020b). Hence, we speculated that SyCsm6 might have ring nuclease activity. To evaluate cOA degradation, we incubated cA4 with SyCsm6 and tested whether the pre-incubated cA4 could activate SyCsx1 in a cleavage assay. Figure 4D shows that after denaturation and removal of SyCsm6, the pre-treated cA4 sample activated SyCsx1 to a much lower extent compared to a cA4 sample receiving only a mock treatment. In contrast, complete SyCsx1 activation and therefore no putative cleavage of cA4 was observed when the cyclic molecule was pre-treated with the SyCsm6(K167A) variant. This lysine residue is thought to be crucial for cA4 binding. This is in line with data from *Sulfolobus solfataricus* showing that the corresponding mutation in Sso1393 leads to a catalytic inactive enzyme (Athukoralage et al., 2018). To further validate that the differences in SyCsx1 activation are linked to cA4 degradation, the assays were repeated with the cA4 analog tetrafluoro-c-tetraAMP (cFA4) that is resistant to cleavage. Overall, cFA4 is a less potent activator of SyCsx1 as seen by the slower substrate turnover (Figure 4E). This is consistent with data showing that the 2′-fluoro modifications in hexafluoro-c-hexaAMP (cFA6) lead to a weaker activation of Csm6 from *Enterococcus italicus*, likely due to lower binding affinity of the modified activator (Garcia-Doval et al., 2020). Importantly, pre-incubation with active SyCsm6 decreases the ability of cFA4 to activate SyCsx1 much less when compared to unmodified cA4 implying that the observed effect is caused by cOA cleavage.

To identify potential cleavage products, we analyzed the turnover of cOAs by liquid chromatography coupled with high-resolution mass spectrometry. After incubating cA4 for 120 min with SyCsm6, the substrate peak was gone and cA4 was converted to a product with a retention time of 7.8 min. This was identified as a linear diadenylate with a cyclic 2′,3 terminus (A2 > P), as we were able to show high agreement in the fragment pattern as well as in the exact mass, drift time, collision cross-section (CCS) value and retention time compared to a standard (Supplementary Figure S2). Similarly, cA6 was cleaved to A2 > P and an additional product. This is likely a triadenylate as it has the exact mass as well as drift time and CCS value as a c-tri-AMP standard (Supplementary Figure S3). However, it differs in both the retention time and fragment spectra and we speculate that it is the linear form carrying a 2′,3′-cyclic phosphate group (A3 > P). No accumulation of these cleavage products of cA4 or cA6 were observed when these substrates were incubated with the SyCsm6(K167A) mutant (Supplementary Figure S4). Together, these results indicate that SyCsm6 catalyzes cOA cleavage producing the same linear An>P forms that are generated by the CARF domain of stand-alone ring nucleases and self-inactivating Csm6 homologs (Athukoralage et al., 2018; Garcia-Doval et al., 2020; Smalakyte et al., 2020).

## Discussion

A new mechanism by which CRISPR systems provide immunity against invading genetic elements has emerged in the past few years. Studies in different bacteria and archaea have shown how type III CRISPR-mediated cOA signaling activates ancillary nucleases that enhance antiviral defense and how enzymes with ring nuclease activity cleave cOA to switch off these nonspecific nucleases to facilitate cell recovery (Athukoralage and White, 2021). To our knowledge, cOA signaling *via* type III CRISPR systems was not shown for cyanobacteria yet, though these phototrophic bacteria are very rich in various type III CRISPR systems (Makarova et al., 2015; Shah et al., 2019). Here we characterized two ancillary CRISPR proteins in a cyanobacterial model organism and showed that cyanobacteria encode proteins that harbor enzymatic activities required for cOA-mediated defense against invading genetic elements.

SyCsx1 is a CARF-family effector protein harboring a HEPN domain with RNase activity specifically activated by cA4 but not by cA6 (Figures 2C–E). While we cannot exclude activation by cyclic nucleotides of different sizes, most characterized CARF domain effectors specifically recognize one of these two second messengers (Kazlauskiene et al., 2017; Niewoehner et al., 2017; Rouillon et al., 2018; Molina et al., 2019; Garcia-Doval et al., 2020; McMahon et al., 2020). Although there is no experimental evidence that cA4 is synthesized by the Cas10 homolog of the CRISPR2 (III-D) system in *Synechocystis*, sequence analysis indicates that it harbors conserved Palm1 and Palm2 domains which are required for cOA synthesis (Supplementary Figure S5; Kazlauskiene et al., 2017; Niewoehner et al., 2017; You et al., 2019; Jia et al., 2019a). In addition, it is plausible that the Cas10 homolog of the CRISPR3 (III-B) system generates cOAs. Once activated, SyCsx1 specifically degrades single-stranded RNA with a stark preference for phosphodiester bonds involving cytosine nucleotides (Figure 3). Such nucleotide-specificity is known from other Csx1/Csm6-family effectors. For example, Molina et al. (2019) reported that SisCsx1 exhibits a strong preference for cleaving 5’-C-C-3′ dinucleotides. Other effectors like SthCsm6 and *Staphylococcus epidermidis* Csm6 showed a preference for cleaving after purines (Kazlauskiene et al., 2017; Foster et al., 2019) or are specific for adenosines (e.g., PfuCsx1 and TonCsm6; Sheppard et al., 2016; Jia et al., 2019b). Considering that SyCsx1 favors cytosine residues but cuts an ssRNA substrate that contains no long C-stretches, we can assume that SyCsx1 cuts before/after cytosine or in between CC dinucleotides. Favoring only one or two nucleotides makes SyCsx1 a mostly sequence-unspecific endoribonuclease that will target most cellular RNAs. We can only speculate that the observed nucleotide preference of SyCsx1 and other effectors is related to a difference in base composition of typically encountered invading genetic elements and essential host mRNAs. As the Cas10 homolog of CRISPR2 is missing the HD nuclease domain, which is cleaving single-stranded DNA during interference in canonical type III systems, it is tempting to speculate that the CRISPR2 system might depend on SyCsx1 activity for clearing invader sequences that are not complementary to the targeting crRNA. *S. solfataricus* Csx1 which binds cA4 with a dissociation constant (KD) of 130 ± 20 nM has a multiple-turnover kinetic constant (*k*_cat_) for cA4-activated RNA cleavage of 0.44 ± 0.03 min^−1^ (Athukoralage et al., 2020b). As we did not aim at measuring kinetic parameters of SyCsx1 activity in detail, we are not able to compare the activities of both proteins directly. However, we do not have evidence that the cyanobacterial Csx1 behaves very different from the archaeal one.

Our results clearly demonstrate that SyCsm6 has ring-nuclease activity and can degrade cOAs, similar to other Csm6 family RNases that have a CARF domain and act as a dual cOA sensor and ring nuclease (Athukoralage et al., 2019; Jia et al., 2019b; Garcia-Doval et al., 2020; Smalakyte et al., 2020). While SyCsx1 is specifically activated by cA4, the CARF domain of SyCsm6 is apparently less specific as it binds and cleaves cOAs of various lengths. Such indiscriminate activity by the CARF domain was not observed in a previous study on Csm6 from *Streptococcus thermophilus* which showed specificity for cA6 (Smalakyte et al., 2020). The homology of the SyCsm6 CARF domain to Crn1-like ring-nucleases (Figure 4A) and the appearance of typical An>P cleavage products (Supplementary Figure S3) suggest that the CARF-domain catalyzes the cleavage of its cOA ligands. Nonetheless, we cannot definitely exclude a role of the RelE domain in cOA cleavage. As the CRISPR2 (III-D) system in *Synechocystis* does not encode any other obvious ring nuclease, SyCsm6 activity is likely the main “off-switch” that controls SyCsx1 activity by eliminating excess cOAs.

The absence of *in vitro* RNase activity in SyCsm6, should not be generalized. Considering the ribosome-dependent cleavage mechanism of *E. coli* RelE (Christensen and Gerdes, 2003; Pedersen et al., 2003; Neubauer et al., 2009), one could speculate that the SyCsm6 RelE domain can degrade actively translated mRNAs *in vivo* in a cOA-dependent manner. Nonetheless, this is purely speculative, as no CARF-RelE domain proteins have been characterized *in vivo* so far. Taken together, SyCsm6 is possibly a bifunctional, self-inactivating effector protein that minimizes self-toxicity.

Gradient profiling experiments suggest that SyCsx1 and SyCsm6 are part of the same complex (Riediger et al., 2021). Both proteins were shown to sediment together with crRNAs and ancillary proteins from the CRISPR2-system after separation of a whole cell extract in sucrose density gradient centrifugation. Hence, we suspect that in *Synechocystis*, the CARF-domain proteins are in close proximity to each other and to the Cas10 effector complex. Such a close association could facilitate invader clearance by ensuring timely activation and termination of cOA signaling and spatially limiting unspecific RNase activity.

The CRISPR2 (III-D) system of *Synechocystis* harbors required enzymatic functions for a functional cA4-mediated defense mechanism. Intriguingly, the CRISPR3 (III-B) system is a putative source of additional cOA synthesis and encompasses a gene (*slr7080*) that encodes a putative metal-dependent Csx3/Crn3 ring-nuclease domain (Slr7080) fused to an AAA+ ATPase domain with an uncharacterized cellular function (Shah et al., 2019; Athukoralage et al., 2020a). While both systems could rely on mutual exclusive cOAs, there is the potential for cross-talk between both systems to coordinate defense against invading genetic elements.

## Materials and methods

### Heterologous overexpression and purification of SyCsx1 and SyCsm6 in *Escherichia coli*

The gene sequences of *slr7061* (SyCsx1) and *sll7062* (SyCsm6) were amplified from *Synechocystis* genomic DNA, ligated into the pQE-80 vector by T4 DNA Ligase (NEB), and propagated in *E. coli* DH5α competent cells. Variants of SyCsx1 and SyCsm6 were generated by site-directed mutagenesis using fast cloning (Li et al., 2011) and verified by sequencing. Primers used in this study are shown in Supplementary Table S1. For overexpression of His-tagged proteins, BL21 (DE3) *E. coli* cells were transformed with the respective plasmids. Expression was induced with 1 mM isopropyl β-D-1-thiogalactopyranoside (IPTG) at an OD_600nm_ of ~0.6. Proteins were expressed overnight at 18°C in the case of SyCsx1 variants and 25°C for SyCsm6 variants. Cells were harvested by centrifugation at 4,000 × g at 4°C for 15 min, and pellets were resuspended in lysis buffer (50 mM Tris pH 8.0, 150 mM NaCl) supplemented with protease inhibitors (4 mM p-aminobenzamidine and 40 mM 6-aminohexaonic acid), and subsequently lysed by a French press. Cell lysates were centrifuged at 20,000 × g for 30 min at 4°C, and supernatants were filtered and loaded on Ni-NTA gravity columns (QIAGEN). After washing with lysis buffer supplemented with 10 mM imidazole, proteins were eluted with lysis buffer containing 200 mM imidazole. For further purification, the elution fractions were applied to size exclusion chromatography with a Superdex 200 10/300 column (GE Healthcare) in lysis buffer supplemented with 5% glycerol. Finally, all proteins were concentrated, aliquoted, flash-frozen with liquid nitrogen, and stored at −80°C.

### Gel-based cleavage assays

Synthetic RNA and DNA oligos used in this study are listed in Supplementary Table S1. To hybridize dsRNA and dsDNA substrates, complementary oligos (RNA1/RNA2, DNA1/DNA2) were mixed in a 1:1 molar ratio in annealing buffer (10 mM HEPES pH 7.5, 50 mM NaCl, and 1 mM EDTA), heated for 5 min at 95°C, and cooled down (1°C per minute) to 25°C. Substrate specificity was determined by incubating 300 nM SyCsx1 with 5 μM substrate in reaction buffer (50 mM HEPES pH7.5, 50 mM KCl, 1 mM DTT, 300 nM cA4) at 30°C. The reaction was stopped at the indicated timepoints by adding 2 × RNA loading dye (New England Biolabs). To investigate sequence specificity, 25 nt RNA homoribopolymers (800 pmol with the exception of 25 pmol for poly(G)) were cleaved in the same way. 20 μl of the samples were heated for 10 min at 65°C and then separated by 12% PAA gel in 1× TBE (100 mM Tris borate pH 8.3, 20 mM EDTA,) at 120 V for 2 h. Nucleic acids were visualized using either SYBR Gold or ethidium bromide.

### Fluorescence-based SyCsx1 activity assay

To measure SyCsx1 activity, 300 nM SyCsx1 or its mutant variants were mixed with 300 nM cA4 (Biolog Life Science Institute GmbH & Co. KG, Germany) in reaction buffer in a total volume of 100 μl. The mix was heated to 30°C and the reaction was started by adding 40 nM of RNaseAlert substrate (Integrated DNA Technologies). The fluorescence signal (excitation at 490 nm, emission at 520 nm) was continuously measured in a TECAN Infinite 200 plate reader.

The influence of SyCsm6 on SyCsx1 activity was determined by pre-incubating 20 nM cA4 or cFA4 (Biolog Life Science Institute GmbH & Co. KG, Germany) with 500 nM SyCsm6 or its mutant variant in reaction buffer at 30°C for 60 min. The reaction was stopped by heating the sample to 95°C for 5 min and denatured SyCsm6 was removed by centrifugation (20,000 × g for 5 min). Subsequently, the sample was used to activate the cleavage of 20 nM RNaseAlert substrate by 300 nM SyCsx1 in a total volume of 100 μl as before.

### LC–MS analysis of cOA degradation products

The degradation of cA4 or cA6 (final concentration of 667 nM) was assayed by incubation of the substrates with 2 μM SyCsm6 or the K167A mutant at 30°C for 2 h in a total volume of 300 μl. The degradation products were extracted in 1.2 ml extraction solution (50:50 (v/v) acetonitrile and methanol, HPLC grade). All samples were incubated for 15 min on ice, then heated to 95°C for 10 min, and immediately placed on ice. Precipitated proteins were removed by centrifugation (20,000 × g, 10 min at 4°C). The resulting final supernatants were dried in a speed-vac at 42°C. The residual pellet was resolved in 200 μl HPLC grade water (J.T. Baker, Deventer, The Netherlands). Then, 40 μl of the sample was mixed with 40 μl of water containing the internal standards (200 ng/ml ^13^C_2_0^15^N_10_-c-di-GMP, 200 ng/ml ^13^C_2_0^15^N_10_-c-di-AMP, and 100 ng/ml Tenofovir) and transferred to measuring vials.

For the identification of the cA4 and cA6 products, a LC–MS-IMS-qTOF experiment was performed on a ACQUITY UPLC I-Class/Vion IMS-QTOF high resolution LC–MS system (Waters Corporation, Milford, MA, USA). Therefore, a C18 column (Nucleodur Pyramid C18 3 μ 50 × 3 mm; Macherey Nagel, Düren, Germany) connected to a C18 security guard (Phenomenex, Aschaffenburg, Germany) and a 2 μm column saver was used. The column was kept at 50°C. A binary gradient of water containing 10 mM ammonium acetate (solvent A) and methanol (solvent B) was applied to achieve chromatographic separation of the analytes using a flow rate of 0.4 ml/min during the whole chromatographic run. The eluting program was as follows: 0 to 4 min: 0% B, 4 to 7.3 min: 0 to 10% B. This composition was hold for 1 min. Then the organic content was increased to 90% within 10.7 min followed by a 6 min re-equilibration step with 0% B. Total analysis run time was 25 min. High resolution mass spectrometry data were collected on a Vion IMS-QTOF mass spectrometer equipped with an electrospray ionization source (ESI). The ESI was operating in positive ionization mode using a capillary voltage of 2.5 kV and the cone voltage of 40 V. The source temperature and desolvation gas temperature was set at 150°C and 600°C, respectively. Analyte fragmentation was achieved using nitrogen as collision gas. Collision energy of 10 V was used to obtain a low collision energy spectrum. For high collision energy spectrum, the collision energy was ramped from 21 to 42 V. Mass to charge ratios between 50 and 2000 were collected. Data acquisition was controlled by the UNIFI 1.9.4.0 software (Waters). For metabolite identification, the retention times, drift times, CCS value and fragment spectra of a c-tri-AMP- and a A(3′,5′)pA(2′,3′)cp-standard were collected as a reference and compared to those of the suspected products in the samples.

## Data availability statement

The original contributions presented in the study are included in the article/Supplementary materials, further inquiries can be directed to the corresponding author.

## Author contributions

AW, NS, JD designed the study. JD and NS performed experiments, analyzed the data and wrote the first draft of the manuscript. HB conducted the LC–MS analysis. All authors contributed to the article and approved the submitted version.

## Funding

This work was supported by the Deutsche Forschungsgemeinschaft (grant AW 2014/9-1 to AW) within the priority program “Much more than defense: The multiple functions and facets of CRISPR-Cas” (SPP2141). We acknowledge support by the Open Access Publication Fund of the University of Freiburg (Germany).

## Conflict of interest

The authors declare that the research was conducted in the absence of any commercial or financial relationships that could be construed as a potential conflict of interest.

## Publisher’s note

All claims expressed in this article are solely those of the authors and do not necessarily represent those of their affiliated organizations, or those of the publisher, the editors and the reviewers. Any product that may be evaluated in this article, or claim that may be made by its manufacturer, is not guaranteed or endorsed by the publisher.
