# Supplementary material for: Enzymatic properties of CARF-domain proteins in Synechocystis sp. PCC 6803
Source: Front Microbiol. 2022 Nov 7;13:1046388. doi: 10.3389/fmicb.2022.1046388 (PMC9676260; doi:10.3389/fmicb.2022.1046388)
Supplement: Supplementary file 1 [file Data_Sheet_1.PDF]

Supplementary Material

Table S1: Oligonucleotides used in this work.

Name	Description	Sequence
Csx1 (BamHI)-fw	Forward primer for Csx1 cloning into pQE-80	GGATCCATGAAAATCATCTCTTTTCTCG
Csx1 (KpnI)-rev	Reverse primer for Csx1 cloning into pQE-80	GGTACCTTACTGCTTGAGTAATGAATTG
Csm6 (BamHI)-fw	Forward primer for Csm6 cloning into pQE-80	GGATCCCCTAACGTTATTGTTTCCCCTTG
Csm6 (KpnI)-rev	Reverse primer for Csm6 cloning into pQE-80	GGTACCTTATAAATGCAGCCCCAATTTATC
Csx1-D114A-fw	Forward primer for Csx1_D114A mutation	TCTTTGCCTTTACCCATGGTTATCGTTTTTTAC
Csx1-D114A-rev	Reverse primer for Csx1_D114A mutation	GATAACCATGGGTAAAGGCAAAGATAACTCGATC
Csx1-H117D-fw	Forward primer for Csx1_H117D mutation	CTTTACCGACGGTTATCGTTTTTTACCAG
Csx1-H117D-rev	Reverse primer for Csx1_H117D mutation	AACGATAACCGTCGGTAAAGTCAAAGATAAC
Csx1-G118A-fw	Forward primer for Csx1_G118A mutation	TTGACTTTACCCATGCCTATCGTTTTTTACC
Csx1-G118A-rev	Reverse primer for Csx1_G118A mutation	ATAGGCATGGGTAAAGTCAAAGATAACTCG
Csx1-R120A-fw	Forward primer for Csx1_R120A mutation	TTTACCCATGGTTATGCCTTTTTACCAGTG
Csx1-R120A-rev	Reverse primer for Csx1_R120A mutation	AAGGCATAACCATGGGTAAAGTCAAAGATAAC
Csx1_H388A_fw	Forward primer for Csx1_H388A mutation	CTTGCCGCCTGTGGTATGAGAAAAGATCCCAAATC
Csx1_H388A_rev	Reverse primer for Csx1_H388A mutation	TTCTCATACCACAGGCGGCAAGGTCGTTGCGAATTTC
Csm6_K167A_fw	Forward primer for Csm6_K167A mutation	GATTTGCTAGCGTTAATGGCTTCCTGC
Csm6_K167A_rev	Reverse primer for Csm6_K167A mutation	GCCATTAACGCTAGCAAATCCTCCAG
RNA1	ss RNA (+)	GCAACACUAAGCGGCGUCGAGGGCGAGAGGACCAAACGA[illegible]
RNA2	ss RNA (-)	GUCGUUUGGUCCUCUCGCCCUCGACGCCGCUUAGUGUUG
DNA1	ss DNA (+)	ACCATCACGGATCCATGGTTAGTTATCATTTGCACCTGAC
DNA2	ss DNA (-)	GTCAGGTGCAAATGATAACTAACCATGGATCCGTGATGGT
poly(A)	Poly-adenine$_{(25)}$	AAAAAAAAAAAAAAAAAAAAAAAAA
poly(U)	Poly-uridine$_{(25)}$	UUUUUUUUUUUUUUUUUUUUUUUUU
poly(C)	Poly-cytosine$_{(25)}$	CCCCCCCCCCCCCCCCCCCCCCCCC
poly(G)	Poly-guanine$_{(25)}$	GGGGGGGGGGGGGGGGGGGGGGGGG

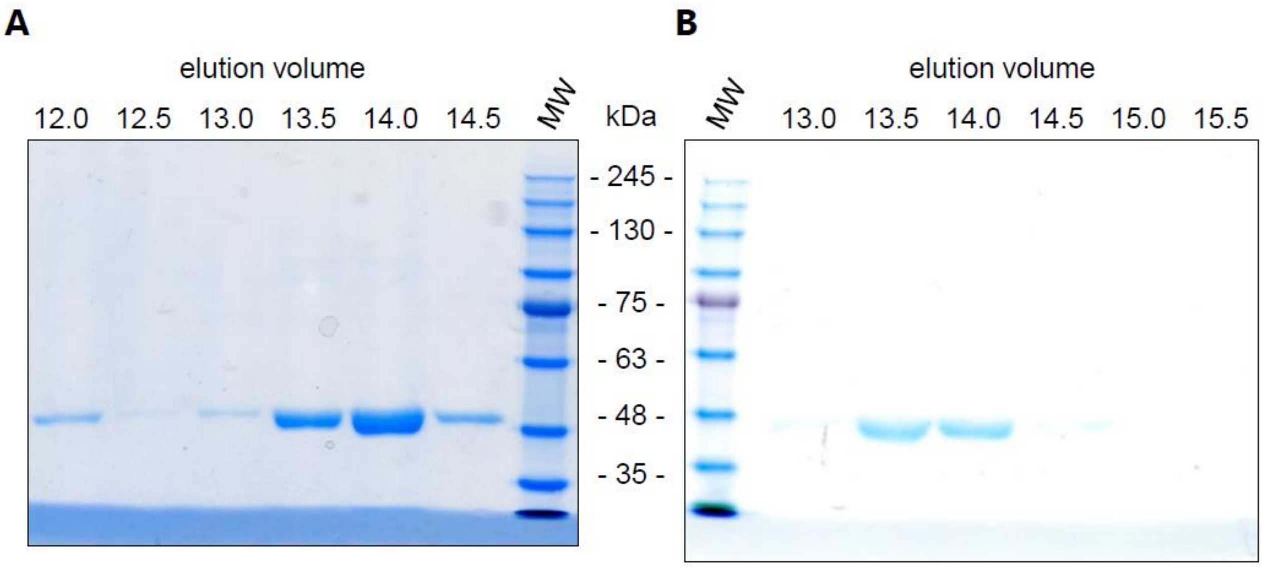

Figure S1: Elution fractions after affinity and size-exclusion chromatography. (A) His-tagged SyCsx1 (48.7 kDa) and **(B)** His-tagged SyCsm6 (43.5 kDa) were isolated from *E. coli* by affinity chromatography and further purified by size-exclusion chromatography on a Superdex 200 10/300 column (upper panel). Elution fractions were analyzed on a 10% SDS-PAGE and stained with Coomassie Brilliant Blue.

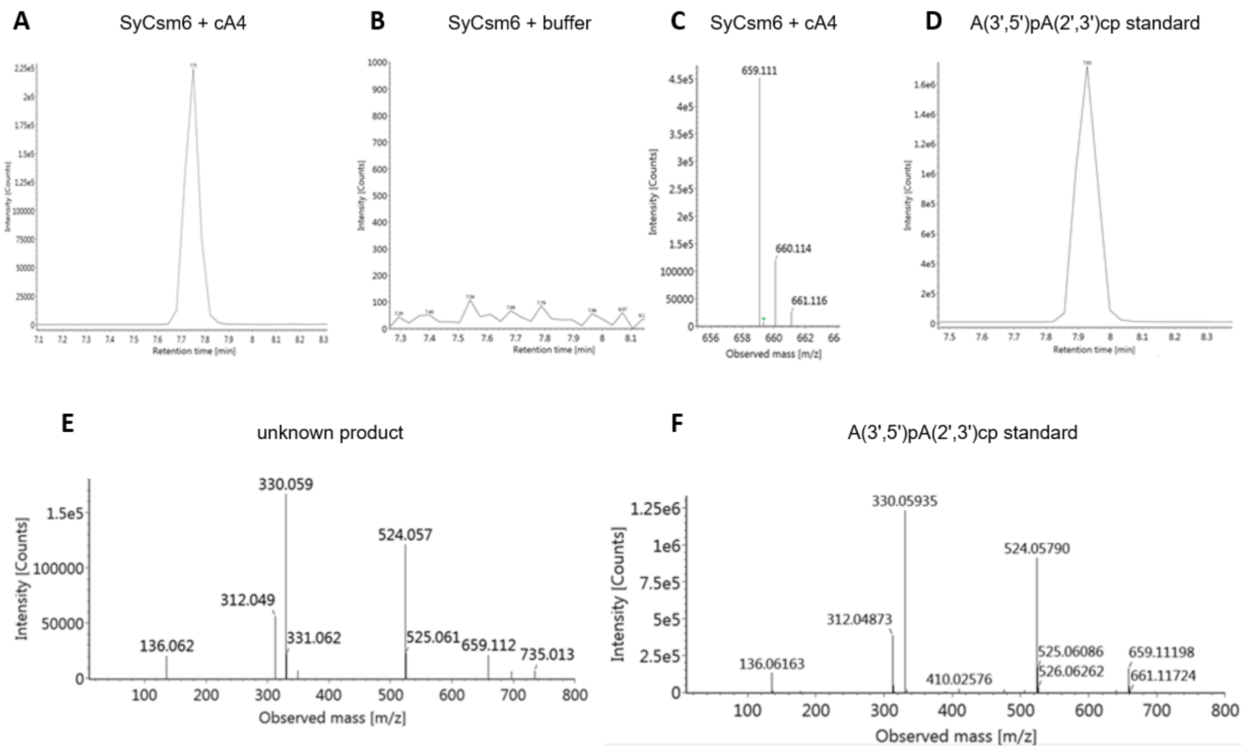

Figure S2: Analysis of cA4 degradation by SyCsm6: 667 nM cA4 was incubated with 2 µM SyCsm6 at 30 °C for 2 h in 300 µl reaction buffer (50 mM HEPES pH7.5, 50 mM KCl, 1 mM DTT). The degradation products were extracted in 1200 µL 50:50 (v/v) acetonitrile/methanol and analysed on a ACQUITY UPLC I-Class/Vion IMS-QTOF high resolution LC-MS system. (**A**) The chromatogram of m/z 659.11 shows a clear signal after 7.8 min when cA4 was incubated with SyCsm6. (**B**) The peak at 7.8 min was not detected in a control missing cA4. (**C**) The isotopic distribution of the signal indicates a single charged ion, resulting in a neutral mass of 658.1 Da. This corresponds to the mass of A(3',5')pA(2',3')cp. (**D**)The control spiked with the presumed product A(3',5')pA(2',3')cp again shows a signal at a comparable retention time. (**E-F**) A comparison of the high energy fragment spectra of m/z 659.11 in the cA4 containing sample and the A(3',5')pA(2',3')cp standard shows high agreement with the prominent fragment ions m/z 136.06, 330.06 and 524.06.

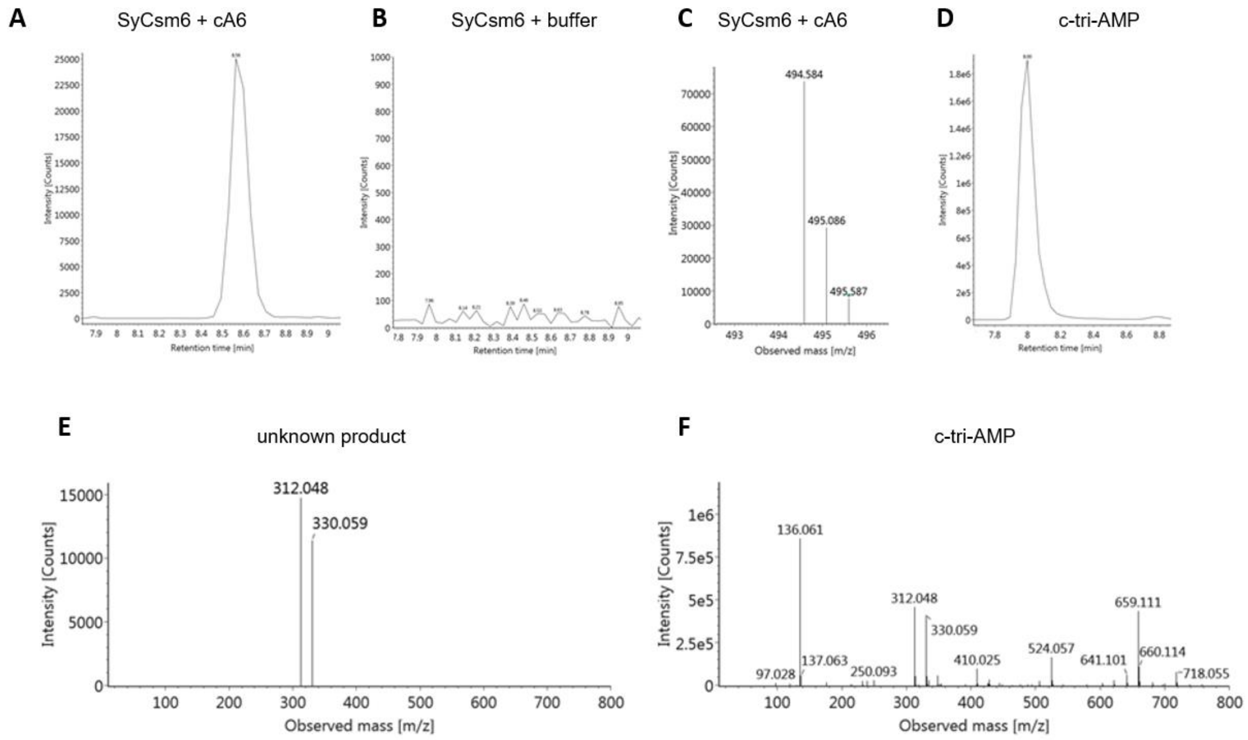

Figure S3: Analysis of cA6 degradation by SyCsm6: 667 nM cA6 was incubated with 2 µM SyCsm6 at 30 °C for 2 h in 300 µl reaction buffer (50 mM HEPES pH7.5, 50 mM KCl, 1 mM DTT). The degradation products were extracted in 1200 µL 50:50 (v/v) acetonitrile/methanol and analysed on a ACQUITY UPLC I-Class/Vion IMS-QTOF high resolution LC-MS system. (**A**) The chromatogram of m/z 494.58 shows a clear signal after 8.0 min retention time when cA6 was incubated with SyCsm6. (**B**) The peak at 8.0 min was not detected in a control missing cA6. (**C**) The isotopic distribution of the signal indicates a double charged ion, resulting in a neutral mass of 987 Da. This corresponds to the mass of c-tri-AMP. (**D**) The control spiked with the c-tri-AMP standard shows a signal at 8.5 retention time. (**E-F**) A comparison of the high energy fragment spectra of m/z 494.58 in the cA6 containing sample and the c-tri-AMP standard shows only a low level of agreement.

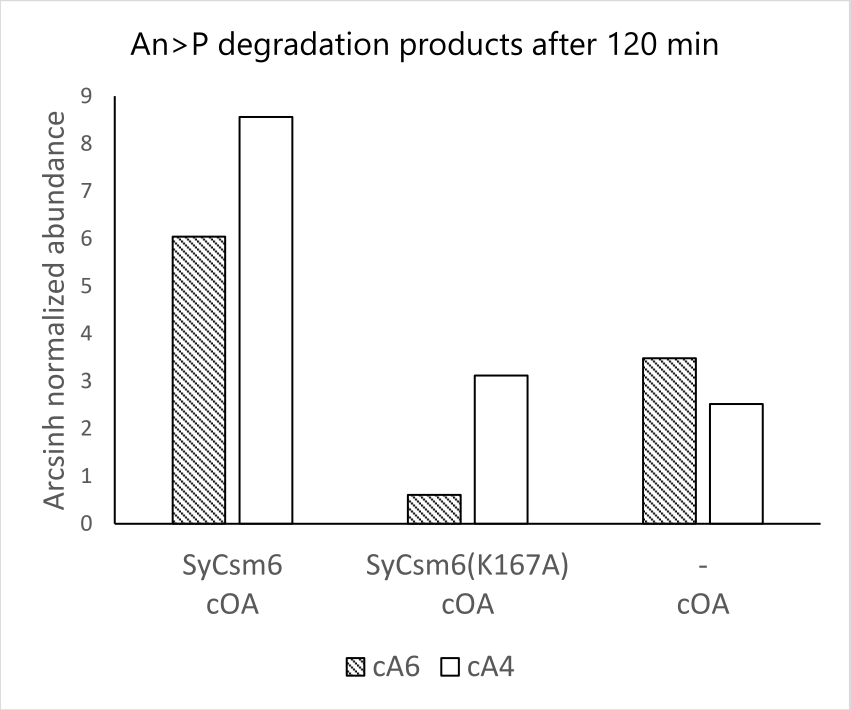

Figure S4: Accumulation of An>P products after cOA degradation by SyCsm6: 667 nM cA6 was incubated with or without 2 µM SyCsm6 at 30 °C for 2 h in 300 µl reaction buffer (50 mM HEPES pH7.5, 50 mM KCl, 1 mM DTT). The degradation products were extracted in 1200 µL 50:50 (v/v) acetonitrile/methanol and analysed on a ACQUITY UPLC I-Class/Vion IMS-QTOF high resolution LC-MS system. The putative An>P products shown in Figure S1 and S2 accumulate in wild type SyCsm6 but not in the SyCsm6(K167A) mutant. Mean abundance values from two experimental replicates are shown.

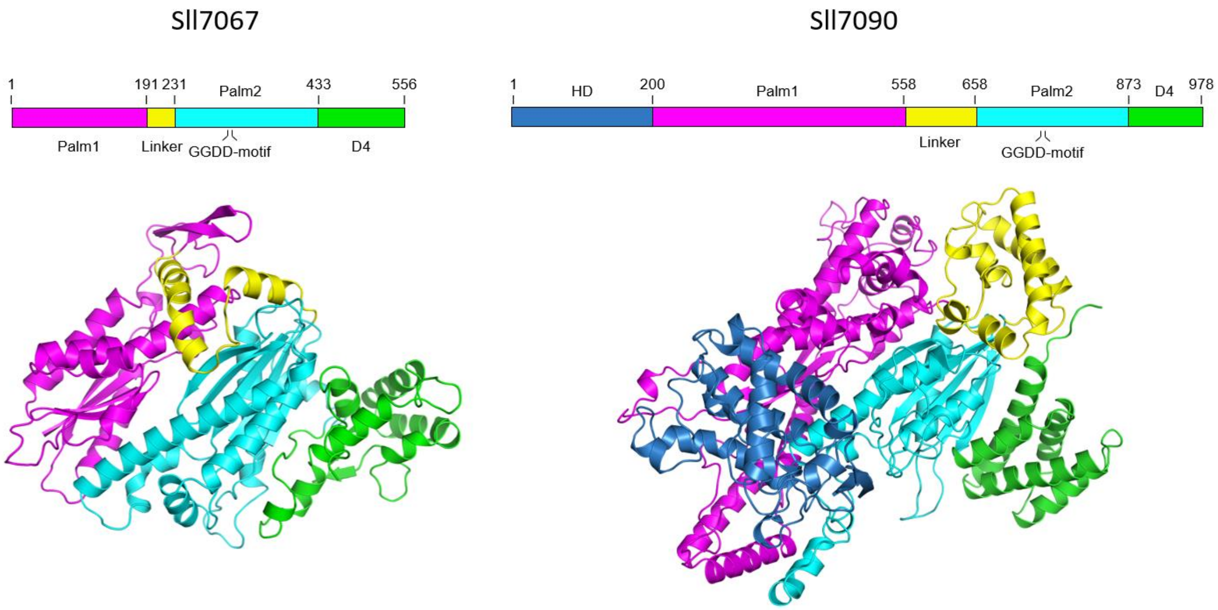

Figure S5: Domain architecture of the Type III CRISPR Cas10/Cmr2 homologs from *Synechocystis* sp. PCC6803. Ribbon representation of the structures of Sll7067 (Q6ZED1) and Sll7090 (Q6ZEA8) as predicted by AlphaFold2 (Jumper et al., 2021). The domain boundaries were estimated by comparing the models to known structures of *P. furiosus* Cmr2 (PDB: 4W8Y) and *T. onnurineus* Csm1 (PDB: 4UW2).

Literature

Jumper, J., Evans, R., Pritzel, A., Green, T., Figurnov, M., Ronneberger, O., et al. (2021). Highly accurate protein structure prediction with AlphaFold. *Nature* 596, 583–589.
